# YY1 and eIF4A3 are mediators of the cell proliferation, migration and invasion in cholangiocarcinoma promoted by circ-ZNF609 by targeting miR-432-5p to regulate LRRC1

**DOI:** 10.18632/aging.203735

**Published:** 2021-12-13

**Authors:** Canghai Guan, Lang Liu, Yuqiao Zhao, Xianhe Zhang, Guanglin Liu, Haicun Wang, Xin Gao, Xiangyu Zhong, Xingming Jiang

**Affiliations:** 1 Department of General Surgery, The 2nd Affiliated Hospital of Harbin Medical University, Harbin 150086, China

**Keywords:** cholangiocarcinoma, circ-ZNF609, miR-432-5p, LRRC1, YY1/eIF4A3

## Abstract

Cholangiocarcinoma is a highly aggressive malignant tumor, and its incidence is increasing all over the world. More and more evidences show that the aberrant expression of circular RNAs play important roles in tumorigenesis and progression. Current studies on the expression and function of circRNAs in cholangiocarcinoma are scarce. In this study, circ-ZNF609 was discovered as a novel circRNA highly expressed in cholangiocarcinoma for the first time. The circ-ZNF609 expression is connected with the advanced TNM stage, lymphatic invasion and survival time in cholangiocarcinoma patients, and can be used as an independent prognostic factor for the patients. Circ-ZNF609 can promote the cholangiocarcinoma cells proliferation, migration and invasion *in vitro*, it can also catalyze the xenograft growth *in vivo*. The promoting effect of circ-ZNF609 on cholangiocarcinoma is achieved via oncogene LRRC1 up-regulation through targeting miR-432-5p by endogenous competitive RNA mechanism. In addition, transcription factor YY1 can bind to the promoter of ZNF609 to further facilitate the transcription of circ-ZNF609. RNA binding protein eIF4A3 can bind to the pre-mRNA of circ-ZNF609 which promotes the circ-ZNF609 circular formation. Overall, YY1/eIF4A3/circ-ZNF609/miR-432-5p/LRRC1 have a significant role in progression of cholangiocarcinoma, and circ-ZNF609 is expected to become a novel biomarker for targeted therapy and prognosis evaluation of cholangiocarcinoma.

## INTRODUCTION

Among the liver malignancies, the incidence of cholangiocarcinoma (CCA) is second only to hepatocellular carcinoma (HCC), which is a malignant tumor of epithelial origin. The overall incidence of cholangiocarcinoma has increased over the past few decades [1–7]. Spain and Asia were reported to have the highest incidence of CCA (2.8 to 3.3 per 100,000), while the highest mortality rates for cholangiocarcinoma are found in Asians (1.4 per 100,000) and American Indians (1.3 per 100,000) [8, 9]. Currently, early screening and diagnosis of cholangiocarcinoma remains a challenge, as most patients with cholangiocarcinoma are asymptomatic in the early stages, the highly desmoplastic, difficult to obtain anatomic location features, which reduces the sensitivity of cytological and pathological diagnosis methods. As a result, most patients with CCA are in advanced-stage [10]. Consequently, the discovery of new molecular abnormality is essential for targeted therapy and prognostic assessment of this highly aggressive tumor.

Currently, the investigation of circular RNAs (circRNAs) is a hotspot in the field of non-coding RNAs. The circRNAs formed a circular structure by linking free 3’- to 5’- ends. These circRNAs are widely identified in mammalian cells and rarely encode proteins [11, 12]. Recent evidences suggest that circRNAs play important roles in the development of several diseases, including osteoarthritis, intervertebral disc degeneration and tumors. It has also been found that circRNAs performed crucial biological functions through numerous mechanisms, including competitive endogenous RNA (ceRNA), RNA binding protein (RBP), gene transcription, and RNA splicing regulatory factors [13–15]. Although circRNAs are emerging as a new research direction in many tumors, little is known about their association with cholangiocarcinoma.

A novel circRNA, circ-ZNF609 (ID: hsa_circ_0000615), derived from zinc finger protein 609 (ZNF609) pre-mRNA, located at chr15: 64791491-64792365 [16, 17]. Circ-ZNF609 was found to be exceptionally up-regulated and carried out diagnostic biomarker roles in breast cancer, renal cancer, colorectal carcinoma, rhabdomyosarcoma, nasopharyngeal carcinoma, gastric cancer and lung adenocarcinoma [18–25]. Nevertheless, the association of circ-ZNF609 with cholangiocarcinoma has not been investigated, so it was necessary to explore the clinical values and biological functions of circ-ZNF609 in cholangiocarcinoma. In this research, qRT-PCR was used to examine the expression of circ-ZNF609 in CCA tissues and adjacent normal tissues. Furthermore, the expression of circ-ZNF609 and the clinicopathological data of the patients were statistically analyzed. In addition, exogenous regulation of circ-ZNF609 expression is then assessed for malignant biological behavior of CCA cells *in vitro* and *in vivo*. Mechanically, circ-ZNF609 can directly target miR-432-5p and inhibit the binding of miR-432-5p to LRRC1, leading to increased expression of LRRC1 protein and ultimately contributing to the proliferation, migration and invasion. YY1, a transcription factor, is overexpressed in a diverse range of tumors, including cholangiocarcinoma [26]. The present study confirmed that YY1 binds to the circ-ZNF609 promoter and enhances transcription. Furthermore, the RNA binding protein eIF4A3 has been shown to be an essential component of RNA splicing [27]. The present data indicated that eIF4A3 can conjugate to pre-mRNA, thereby accelerating circ-ZNF609 circular formation. In summary, circular RNA ZNF609 is involved in the cholangiocarcinoma development, suggesting that it may be a prospective target for therapeutic and prognostic evaluation.

## RESULTS

### Circ-ZNF609 was highly expressed in CCA and was related to the clinicopathological characteristics

**Figure 1:**
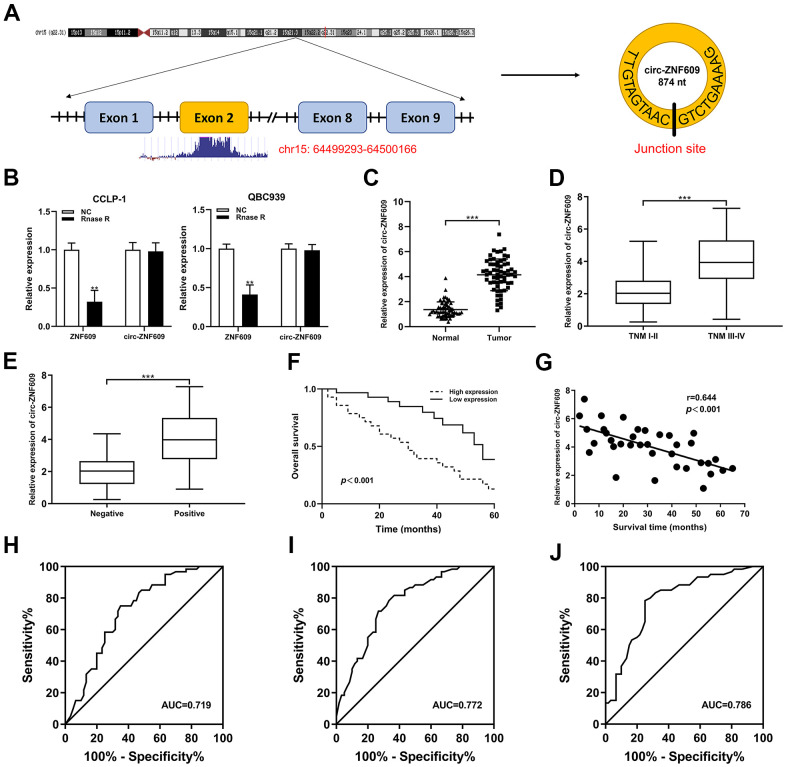
**Circ-ZNF609 was significantly high expression in CCA and associated with clinicopathological characteristics.** (**A**) Schematic representation of circ-ZNF609 genomic structure. (**B**) The circ-ZNF609 and ZNF609 expression were detected through using the qRT-PCR in CCA cells treated with RNase R. (**C**) qRT-PCR was used to detect the relative expression of circ-ZNF609 in CCA tumor tissues and adjacent normal tissues. (**D**) Circ-ZNF609 expression of patients with different TNM stages. (**E**) Circ-ZNF609 expression of patients with or without lymph node metastasis. (**F**) The overall survival of high and low circ-ZNF609 expression group patients. (**G**) The correlation between circ-ZNF609 expression and survival time of CCA patients. (**H**) The ROC curve of circ-ZNF609 detection as a prognostic biomarker. (**I**) The ROC curve of CA19-9 detection as a prognostic biomarker. (**J**) The ROC curve of circ-ZNF609+CA19-9 detection as a prognostic biomarker. **P< 0.01; ***P< 0.001.

Circ-ZNF609 was spliced from exon 2 of ZNF609 (Figure 1A). In Figure 1B, the circ-ZNF609 and ZNF609 expression after using RNase R was detected by qRT-PCR, these results revealed that circ-ZNF609 was more resistant to RNA enzyme digestion than ZNF609. The qRT-PCR data showed that the circ-ZNF609 expression was higher in cholangiocarcinoma tissues than adjacent normal tissues (Figure 1C). The clinicopathological characteristics of CCA patients with circ-ZNF609 revealed that high expression of circ-ZNF609 was significantly associated with advanced TNM and lymph node invasion (Figure 1D, 1E). The association of circ-ZNF609 expression with other clinical parameters is shown in Table 1. Additionally, the overall survival of patients with high circ-ZNF609 expression was inferior (Figure 1F), and circ-ZNF609 was negatively correlated with the survival time of CCA patients (Figure 1G). In addition, anomalous upregulation of circ-ZNF609, lymph node invasion and advanced TNM stage were independent risk factors for unfavorable prognosis in patients with cholangiocarcinoma (Table 2). The receiver operating characteristic curve (ROC) was used to evaluate the prognostic efficiency of circ-ZNF609, CA19-9 and circ-ZNF609+CA19-9 expression. The value of area under curve (AUC) was 0.719 (95% CI: 0.626-0.811), 0.772 (95% CI: 0.688-0.855) and 0.786 (95% CI: 0.703-0.869), respectively (Figure 1H–1J). The results indicated that both circ-ZNF609 and CA19-9 were statistically significant as biomarkers for prognosis evaluation and were more effective predictors when used in combination.

**Table 1:** Correlation between circ-ZNF609 expression and clinicopathological characteristics of cholangiocarcinoma patients.

**Clinicopathological parameters**	**Total cases (n=61)**	**circ-ZNF609 expression**		***P* value**
		**Low (n=30)**	**High (n=31)**	
Age (years)				0.797
<60	25	13	12	
≥60	36	17	19	
Gender				0.795
Male	24	11	13	
Female	37	19	18	
Smoking				0.440
No	26	11	15	
Yes	35	19	16	
Drinking				0.780
No	17	9	8	
Yes	44	21	23	
Histological type				0.335
Adenocarcinoma	49	26	23	
Non-adenocarcinoma	12	4	8	
TNM stage				**0.021**
I-II	33	21	12	
III-IV	28	9	19	
Differentiation grade				0.416
Well/moderate	20	8	12	
Poor/undifferentiated	41	22	19	
Lymph node invasion				**0.002**
Positive	42	15	27	
Negative	19	15	4	
Vascular invasion				0.464
Positive	21	3	6	
Negative	40	21	19	
HBV infection				0.605
Positive	25	11	14	
Negative	36	19	17	
Serum CEA (ng/ml)				0.436
>5	37	20	17	
≤5	24	10	14	
Serum CA19-9 (U/ml)				0.204
>37	32	13	19	
≤37	29	17	12	

**Table 2:** Univariate and multivariate analysis for overall survival of cholangiocarcinoma patients.

**Variables**	**Univariate analysis**			**Multivariate analysis**		
	**HR**	**95% CI**	***P* value**	**HR**	**95%CI**	***P* value**
Age (years)						
≥60 *vs*. <60	1.351	0.781-2.601	0.311			
Gender						
Male *vs*. Female	1.216	0.674-2.360	0.587			
Histological type						
Adenocarcinoma *vs*. Non-adenocarcinoma	1.614	0.852-2.792	0.273			
Differentiation grade						
Poor/undifferentiated *vs*. Well/moderate	1.146	0.642-2.144	0.348			
Vascular invasion						
Positive *vs*. Negative	1.795	0.519-3.235	0.107			
HBV infection						
Positive *vs*. Negative	1.363	0.912-2.348	0.285			
Serum CEA (ng/ml)						
>5 *vs*. ≤5	1.476	0.721-2.783	0.445			
Serum CA19-9 level (U/ml)						
>37 *vs*. ≤37	1.292	0.839-2.433	0.182			
Lymph node invasion						
Positive *vs*. Negative	2.141	1.489-4.317	0.017*	2.284	1.163-3.895	**0.009**
TNM stage						
III-IV *vs*. I-II	2.032	1.301-3.435	0.026*	1.967	0.973-3.252	**0.037**
circ-ZNF609 expression						
Low *vs*. High	2.817	1.843-4.736	0.011*	2.771	1.639-5.379	**0.002**

### Circ-ZNF609 promoted proliferation, migration and invasion of CCA cells

**Figure 2:**
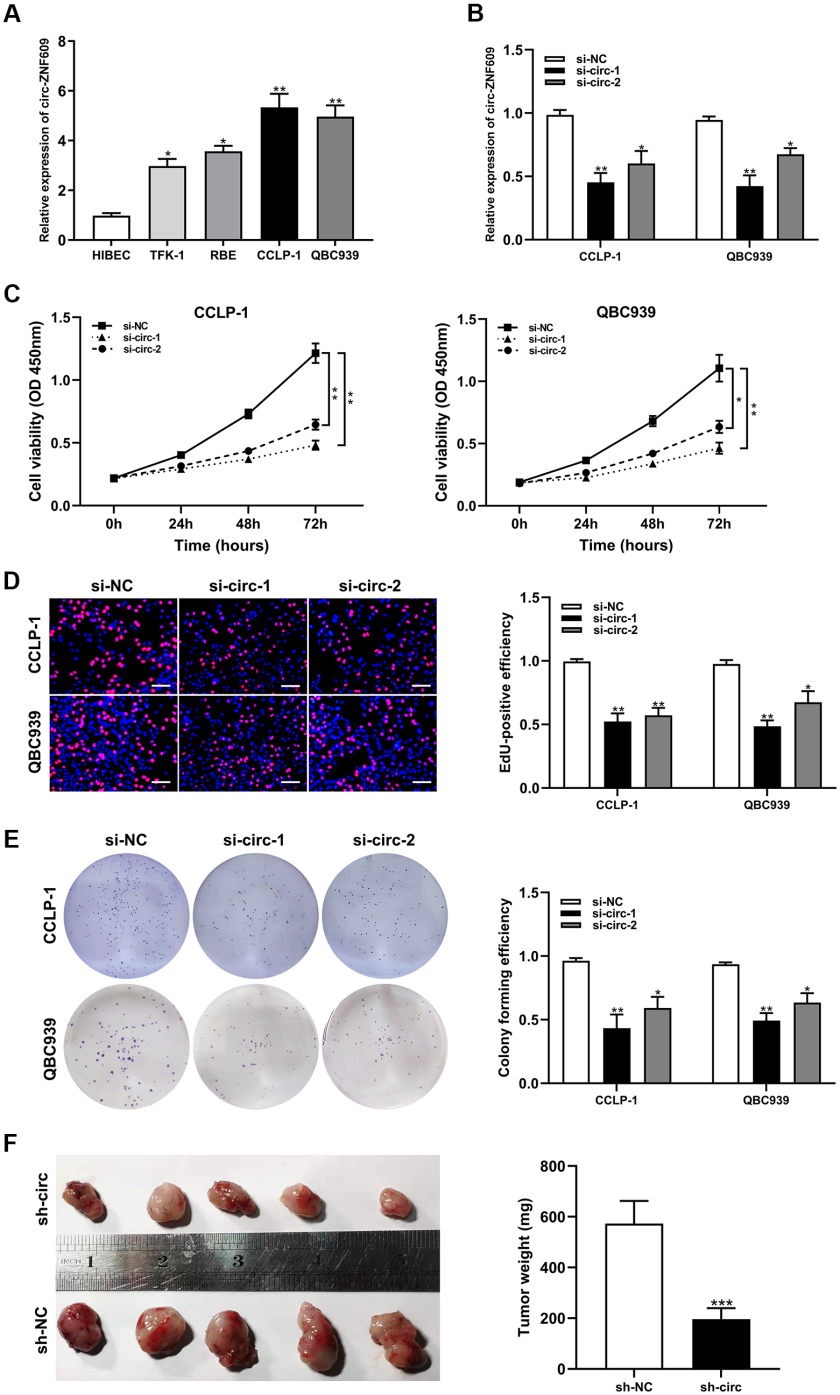
**Silencing circ-ZNF609 inhibited the proliferation of cholangiocarcinoma cells.** (**A**) The relative circ-ZNF609 expression in CCLP-1, QBC939, TFK-1, RBE cells and normal HIBEC. (**B**) The two siRNAs interference efficiency detection of circ-ZNF609. (**C**–**E**) CCK-8 assay, EdU and colony formation assay were used to detect the proliferation ability of CCA cells after circ-ZNF609 knockdown. (**F**) Tumor xenograft assay was used to measure tumor growth after silencing circ-ZNF609 *in vivo*. *P< 0.05; **P< 0.01; ***P< 0.001.

**Figure 3:**
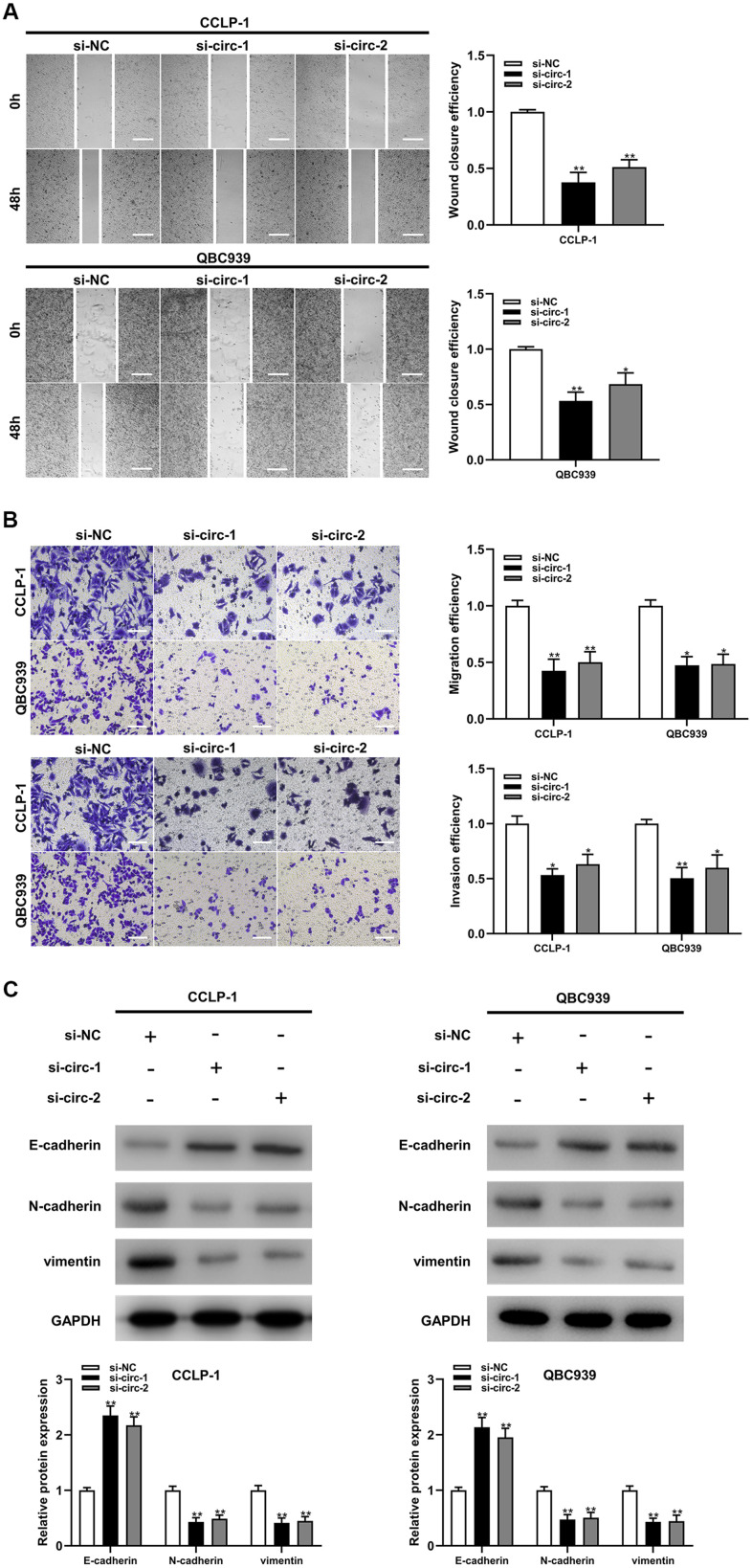
**Silencing circ-ZNF609 inhibited cholangiocarcinoma cells migration and invasion.** (**A**) Wound healing assay was used to detect the migration ability of CCA cells after circ-ZNF609 knockdown. (**B**) The migration and invasive abilities of CCLP-1 and QBC939 were confirmed by transwell assay after silencing circ-ZNF609. (**C**) Western blot was used to detect EMT-related proteins including E-cadherin, N-cadherin and vimentin. **P< 0.01; ***P< 0.001.

Circ-ZNF609 was expressed highly in cholangiocarcinoma cells (TFK-1, RBE, CCLP-1, QBC939) compared to normal biliary cell (HIBEC), and follow-up experiments were performed applying CCLP-1 and QBC939 (Figure 2A). The siRNAs (si-circ-1 and si-circ-2) of circ-ZNF609 both declined the expression of circ-ZNF609 in CCLP-1 and QBC939 cells (Figure 2B). Then, cell viability assays (CCK-8, EdU, colony formation) displayed that knocking down circ-ZNF609 suppressed the CCLP-1 and QBC939 cells proliferation (Figure 2C–2E). In Figure 2F, the growth of mice xenografts was restrained by down-regulation of circ-ZNF609 compared with the control group. The results of wound healing assay suggested that transfection with siRNA-circ-ZNF609 evidently inhibited the migratory ability of CCLP-1 and QBC939 cells (Figure 3A). Transwell assay manifested that the quantity of migrating or invading cells in the lower chamber was considerably reduced after circ-ZNF609 silencing (Figure 3B). Epithelial-mesenchymal transformation (EMT) performed vital roles in tumor metastasis. In Figure 3C, regulating the circ-ZNF609, the expression of E-cadherin, N-cadherin and vimentin was remarkably influenced. It can be speculated that circ-ZNF609, as a pro-cancer circRNA, participated in the CCA proliferation, migration, invasion and EMT process.

### Circ-ZNF609 could sponge miR-432-5p in CCA

**Figure 4:**
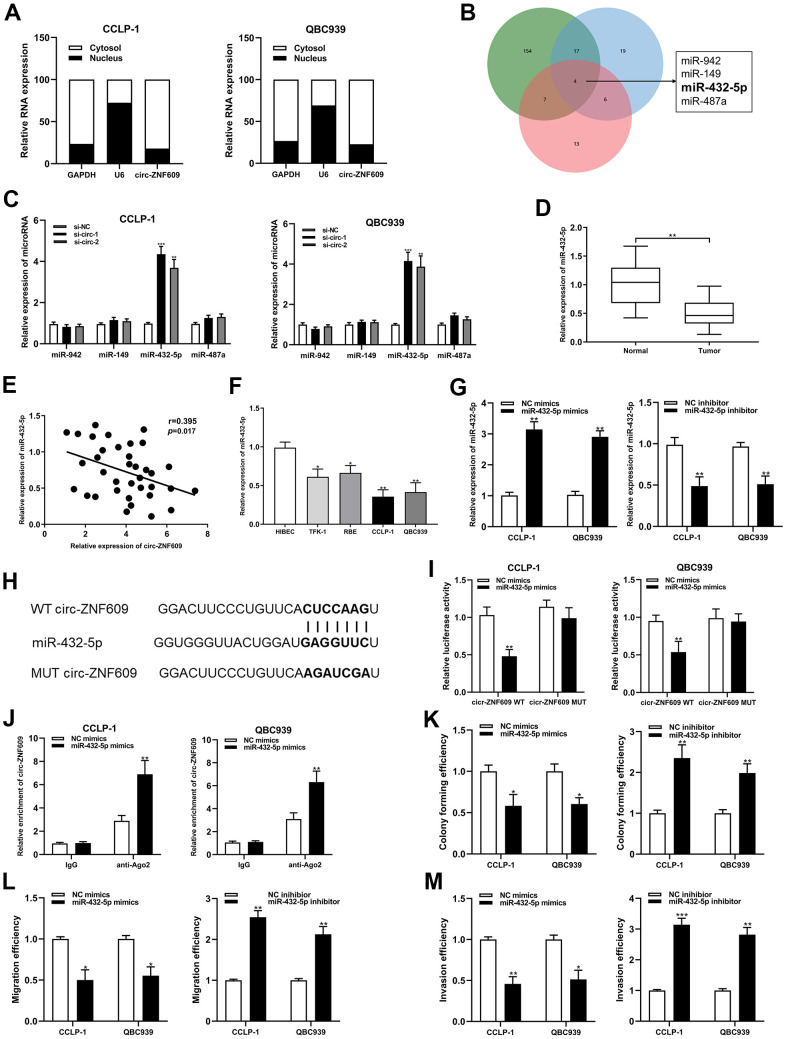
**Circ-ZNF609 was involved in CCA progression via sponging miR-432-5p.** (**A**) Detection of relative expression of circ-ZNF609 in cytoplasm and nucleus of CCA cells by qRT-PCR. (**B**) Targetscan, circinteractome and mirMap were used to identify potential miRNA target genes for circ-ZNF609. (**C**) Relative expression of potential target genes in CCA cells transfected with si-circ-1 and si-circ-2. (**D**) qRT-PCR was used to detect the relative expression of c miR-432-5p in CCA tumor tissues and adjacent normal tissues. (**E**) qRT-PCR was used to detect the correlation between the expression of circ-ZNF609 and miR-432-5p in CCA specimens. (**F**) The relative expression of miR-432-5p in CCA cells detected by qRT-PCR. (**G**) The efficiency detection of miR-432-5p mimics and inhibitor transfection confirmed by qRT-PCR. (**H**) Diagrammatic sketch of the binding sites between miR-432-5p and circ-ZNF609. (**I**) Luciferase activity in CCA cells after transfection of circ-ZNF609 WT or circ-ZNF609 MUT, miR-432-5p mimics or NC mimics. (**J**) RIP assay was utilized to further demonstrate the direct binding between miR-432-5p and circ-ZNF609. (**K**) Colony formation assay was used to detect the proliferation ability after miR-432-5p down-regulation and up-regulation. (**L**, **M**) The migration and invasion changes of tumor cells after miR-432-5p down-regulation and up-regulation detected by transwell. **P< 0.01; ***P< 0.001.

The results of subcellular fractionation assay demonstrated that circ-ZNF609 was predominantly distributed in the cytoplasm (Figure 4A), which indicated that circ-ZNF609 has been involved in post-transcriptional regulation. Extensive studies had confirmed that circRNA can function as a ceRNA to indirectly enhance the expression of downstream target mRNAs by competing with mRNAs to bind miRNAs. On the basis of predictions from bioinformatics databases (targetscan, circinteractome and mirMap) and qRT-PCR detection, miR-432-5p was chosen as the downstream miRNA of circ-ZNF609 (Figure 4B, 4C). MiR-432-5p had low expression in the cholangiocarcinoma tissues and cells, and was negatively correlated with circ-ZNF609 expression (Figure 4D–4F). In Figure 4G, the efficiencies of miR-432-5p mimics and inhibitor identified after transfection into tumor cells were shown. After constructing luciferase reporter plasmid, including wild-type or mutant-type binding sites of circ-ZNF609 and the co-precipitation of RNA with anti-Ago2, the results of dual luciferase reporter assay and RIP assay showed that circ-ZNF609 could directly sponge miR-432-5p (Figure 4H–4J). Up-regulation of miR-432-5p could prohibit the proliferation, migration and invasion ability of CCA cells, and down-regulation of miR-432-5p promoted the proliferation, migration and invasion ability of CCA cells (Figure 4K–4M).

### LRRC1 was the downstream target of miR-432-5p in CCA

**Figure 5:**
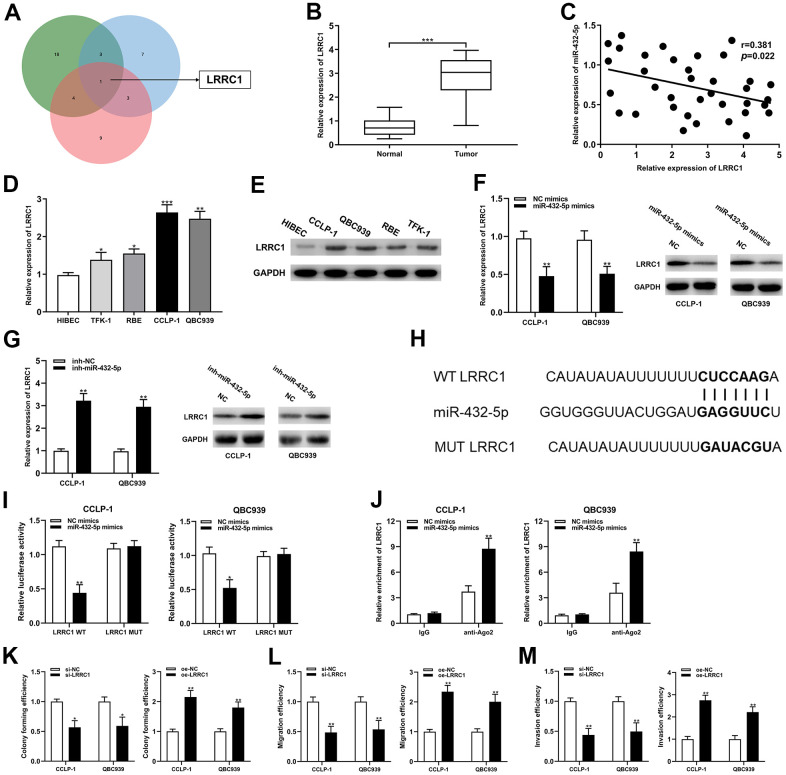
**LRRC1 was a direct target of miR-432-5p in CCA.** (**A**) Targetscan, circinteractome and mirMap were used to identify potential mRNA target genes for miR-432-5p. (**B**) Relative expression of LRRC1 mRNA in CCA tissues and adjacent normal tissues. (**C**) qRT-PCR was used to detect the correlation between miR-432-5p and LRRC1 expression in CCA specimens. (**D**, **E**) The expression of LRRC1 mRNA and protein in CCLP-1 and QBC939. (**F**, **G**) The LRRC1 expression was detected in CCA cells with miR-432-5p up-regulation and down-regulation by qRT-PCR and western blot. (**H**) Diagrammatic sketch of the binding sites between miR-432-5p and LRRC1. (**I**) Luciferase reporter assay in CCA cells transfected with LRRC1 WT or LRRC1 MUT, along with miR-432-5p mimics or NC mimics. (**J**) RIP assay further verified the direct interaction between miR-432-5p and LRRC1. (**K**) Colony formation assay was used to detect the proliferation ability change after LRRC1 down-regulation and up-regulation. (**L**, **M**) The migration and invasion changes of tumor cells after LRRC1 down-regulation and up-regulation detected by transwell. *P< 0.05; **P< 0.01; ***P< 0.001.

The predicted binding sites between miR-432-5p and LRRC1 mRNA 3’-untranslated region (3’-UTR) were determined by bioinformatics databases (targetscan, circinteractome and mirRanda) (Figure 5A). LRRC1 expression was dysregulated in the CCA tumor tissues and cells, and negatively correlated with miR-432-5p (Figure 5B–5E). Increased and decreased miR-432-5p expression could inhibit and LRRC1 mRNA and protein expression, respectively (Figure 5F, 5G). Luciferase activity in CCA cells was confined after co-transfection of LRRC1 wild-type plasmid with miR-432-5p mimics (Figure 5I). Consistently, RIP assay displayed that LRRC1 was distinctly enriched by anti-Ago2 antibodies containing miR-432-5p mimics (Figure 5J). Accordingly, it could be speculated that LRRC1 was a downstream target mRNA of miR-432-5p. In addition, down-regulation of LRRC1 inhibited the proliferation, migration and invasion ability of CCA cells, and up-regulation of LRRC1 promoted them (Figure 5K–5M).

### Circ-ZNF609 regulated CCA malignant biological behavior with miR-432-5p/LRRC1

**Figure 6:**
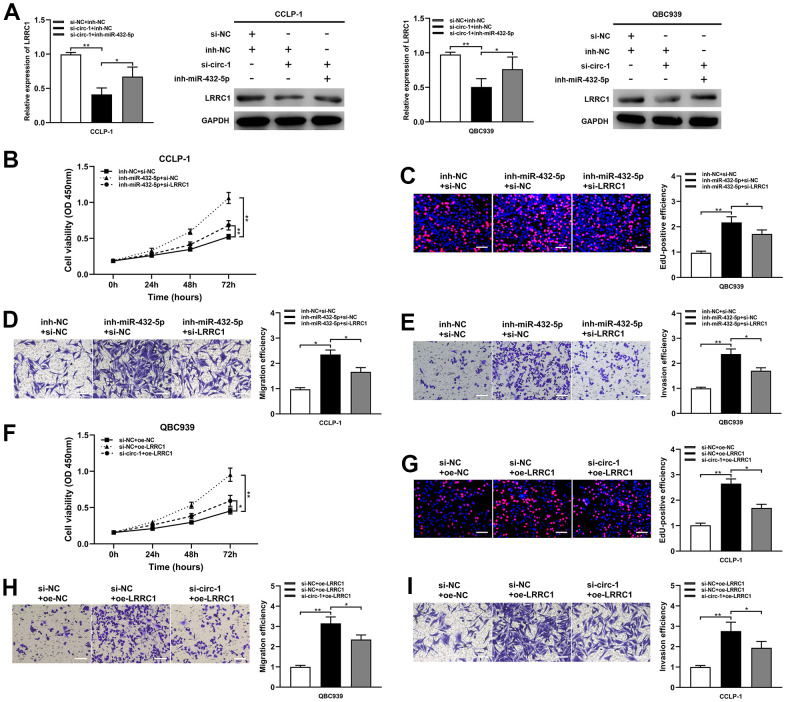
**Circ-ZNF609 up-regulated LRRC1 expression by competitively binding miR-432-5p to induce cholangiocarcinoma development.** (**A**) After co-transfection of circ-ZNF609 siRNA and miR-432-5p inhibitor, the LRRC1 expression was detected by qRT-PCR and western blot. (**B**–**E**) CCK-8, EdU and transwell assays confirmed that the effects of proliferation, migration and invasion induced by miR-432-5p inhibitor were partly reversed by silencing LRRC1. (**F**–**I**) Silencing circ-ZNF609 could partially reverse the proliferation and invasion induced by LRRC1 overexpression in CCK-8, EdU and transwell assays. *P< 0.05; **P< 0.01; ***P< 0.001.

The study data demonstrated that the silencing of miR-432-5p partially reversed the down-regulation of LRRC1 mRNA and protein expression inhibition induced by circ-ZNF609 (Figure 6A). Down-regulation of miR-432-5p on cholangiocarcinoma proliferation, migration and invasion was partially reversed after transfection with si-LRRC1 (Figure 6B–6E). Concurrently, LRRC1 overexpression significantly enhanced tumor cell proliferation, migration and invasion, while knockdown of circ-ZNF609 partially inhibited these inhibitory effects (Figure 6F–6I). These results suggested that circ-ZNF609/miR-432-5p/LRRC1 was involved in tumorigenesis of cholangiocarcinoma.

### YY1 and eIF4A3 could promoted circ-ZNF609 expression

**Figure 7:**
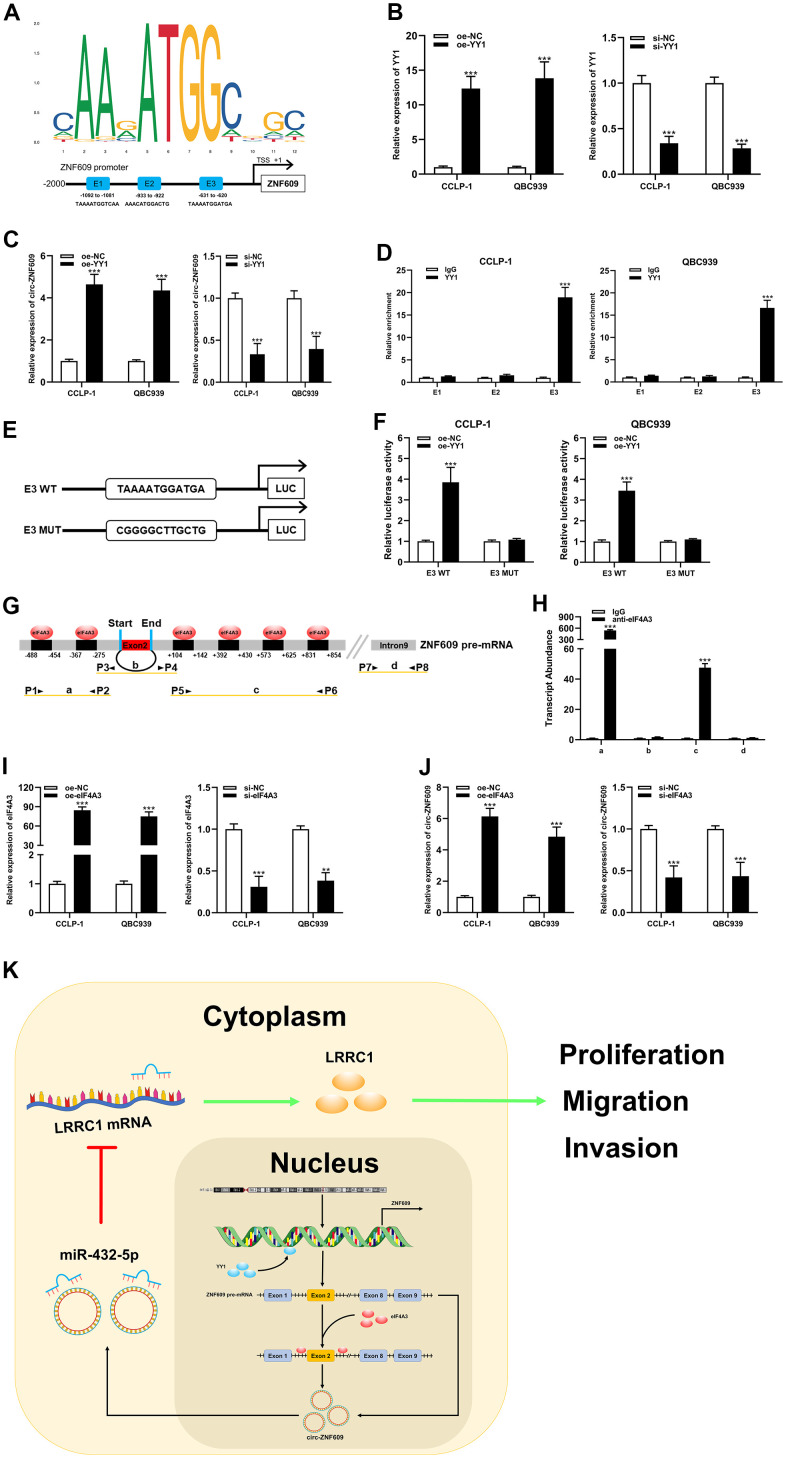
**YY1 and eIF4A3 promoted circ-ZNF609 expression via linking to the circ-ZNF609 promoter and pre-mRNA, respectively.** (**A**) YY1 sequence and binding sites (E1, E2 and E3) to circ-ZNF609 promoter region were predicted by using JASPAR database. (**B**) The efficiency detection of YY1 overexpression and siRNA transfection confirmed by qRT-PCR. (**C**) The circ-ZNF609 expression was detected in CCA with YY1 up-regulation and down-regulation by qRT-PCR. (**D**) ChIP assay was conducted to affirm the direct binding of YY1 to circ-ZNF609 promoter in QBC939 and CCLP-1 cells. (**E**) Schematic diagram of plasmids construction. (**F**) The luciferase activity of E3 wild type was markedly promoted by oe-YY1 co-transfection compared with controls in CCA cells. (**G**) The prediction results of circinteractome database indicated that eIF4A3 had binding sites in the upstream and downstream regions of circ-ZNF609 pre-mRNA. (**H**) The results of RIP demonstrated that eIF4A3 could directly bind to the circ-ZNF609 pre-mRNA in CCA cells. (**I**) Knockdown efficiency and amplification efficiency of eIF4A3 confirmed by qRT-PCR. (**J**) The expression of circ-ZNF609 in CCLP-1 and QBC939 transfected with high expression eIF4A3 and low expression eIF4A3 was detected by qRT-PCR. (**K**) Schematic diagram of circ-ZNF609 tumorigenesis mechanisms in cholangiocarcinoma. **P< 0.01; ***P< 0.001.

For the purpose of exploring the circ-ZNF609 potential upstream regulatory mechanism, screening was performed using the JASPAR database (http://jaspar.genereg.net). As shown in Figure 7A, YY1 is a transcription factor that may bind to circ-ZNF609 promoter, where E1, E2 and E3 are three transcription factor binding sites. To validate this prediction, qRT-PCR results revealed that exogenous regulation of YY1 could influence the expression of circ-ZNF609 directly (Figure 7B, 7C). In addition, the ChIP and luciferase reporter assay results indicated that YY1 was predominantly enriched and bound to the E3 fragments (Figure 7D–7F), which data showed that YY1 could facilitate the transcription of circ-ZNF609 by binding to the E3 region of the ZNF609 promoter. Furthermore, predicted data on circinteractome (https://circinteractome.nia.nih.gov/index.html) indicated that eIF4A3 might combine with circ-ZNF609 pre-mRNA, as shown in Figure 7G. It was previously suggested that the RNA binding protein eIF4A3 was a core component of the exon junction complex and performed RNA splicing functions. The RNA splicing functions important roles in the process of linear RNA forming circular RNA. The binding of eIF4A3 to circ-ZNF609 pre-mRNA was subsequently substantiated by the RIP experimental results (Figure 7H). Moreover, up- and down-regulation of eIF4A3 could respectively enhance and impair the expression of circ-ZNF609 (Figure 7I, 7J). The above results suggested that YY1 could bind to the promoter to further facilitate the transcription of circ-ZNF609 and eIF4A3 could contribute to the circ-ZNF609 circular formation by binding to circ-ZNF609 pre-mRNA (Figure 7K).

## DISCUSSION

Cholangiocarcinoma was an aggressive malignancy, the prevalence of cholangiocarcinoma in Asian countries had increased in the last decades. Survival and prognosis of CCA patients were very unsatisfactory due to difficulties in early diagnosis and monotherapy modalities. Consequently, the discovery and application of new biomarkers for cholangiocarcinoma were the important measure to reverse this situation. CircRNAs were class of back-spliced ncRNAs that form loops and rarely encode proteins. As research progresses, circRNA was no longer considered as by-product of transcription and carried out multiple roles in the normal growth and disease development. For example, circRNA interacted with Pol II transcription complex as a transcriptional regulator, sponged miRNA as ceRNA or recruited protein to activate or repress genes expression [28–30].

Circ-ZNF609 was up-regulated in breast cancer and promoted cell proliferation, migration and invasion through sponging miR-145-5p [18]. Circ-ZNF609 promoted the development of renal carcinoma by the miR-138-5p/FoxP4 axis [19]. The correlation between circ-ZNF609 and cholangiocarcinoma, on the other hand, had not been investigated. In the present study, circ-ZNF609 expression was found to be significantly higher in cholangiocarcinoma tissues and cells than in normal adjacent tissues and cells for the first time. And the dysregulation of circ-ZNF609 was intimately associated with TNM stage, lymph node invasion and prognosis of patients with cholangiocarcinoma. The results of cell viability assay (CCK-8, EdU, colony formation) indicated that down-regulation of circ-ZNF609 inhibited the cholangiocarcinoma cells proliferation. Wound healing and transwell assays showed that silencing circ-ZNF609 impaired the migration and invasion efficiency of tumor cells. The results of western blot demonstrated that knockdown of circ-ZNF609 affected E-cadherin, N-cadherin, vimentin, which suggested that the process of CCA epithelial mesenchymal transformation was restricted. The results of tumor xenograft assay also suggested that exogenous silencing of circ-ZNF609 inhibited tumor growth. This present study confirmed that circ-ZNF609 had a carcinogenic effect in cholangiocarcinoma.

Extensive studies had established that circRNAs could sponge miRNAs through endogenous competitive RNA mechanism and affect the expression of downstream target genes and thus promoted or inhibited occurrence and development of many diseases, including tumors. To illustrate, circGSE1 exerted oncogenic effects in cervical cancer through sponging miR-138-5p [31]. In this study, it was confirmed that circ-ZNF609 could sponge miR-432-5p directly through ceRNA mechanism, and miR-432-5p could further regulate the proliferation, migration and invasion of CCA. Similarly, it was predicted and evidenced that mRNA LRRC1 could be targeted by miR-432-5p in 3’-UTR regions. High expression of LRRC1 promoted malignant biological behaviors of tumor cells in cholangiocarcinoma. Furthermore, the rescue experiments results further demonstrated that circ-ZNF609 affected the proliferation, migration and invasion of CCA by regulating the miR-432-5p/LRRC1 axis.

For the sake of investigating the upstream regulatory mechanisms of circ-ZNF609 in the development of CCA, the transcription factor YY1 with high binding score to ZNF609 promoter region was predicted by using bioinformatics databases. Also, YY1 had been shown to be involved in various tumors progression and associated with adverse clinical prognosis in tumor patients, for instance, YY1 stimulated LINC00667 transcription that enhanced the proliferation of cholangiocarcinoma [26]. The results of present research indicated that elimination of YY1 significantly limited the expression of circ-ZNF609, whereas overexpression of YY1 promoted the circ-ZNF609 expression, which was significantly enriched in the E3 region of circ-ZNF609 promoter. In addition, eIF4A3, as an RNA-binding protein, was a core component of exon junction complex and was recognized as a vital regulator of post-transcriptional regulation, such as mRNA splicing, transport and translation [27]. RNA splicing was one of the essential causes of the circular RNA formation. In this study, it was confirmed by *in vitro* experiments that eIF4A3 could bind to the circ-ZNF609 pre-mRNA to its circular formation, and circ-ZNF609 expression was up- or down-regulated after promoting and inhibiting eIF4A3 expression.

Collectively, this study demonstrated for the first time that the circular RNA ZNF609 was up-regulated in cholangiocarcinoma, its expression was intimately associated with the clinicopathological characteristics and prognosis of patients, and could be an independent prognostic factor for CCA patients. It was further experimentally proved that circ-ZNF609 could facilitate the tumor cells proliferation, migration and invasion in cholangiocarcinoma. Mechanically, circ-ZNF609 sponged miR-432-5p through the ceRNA mechanism, which further strengthened the expression of LRRC1, the downstream oncogene of miR-432-5p. In addition, the upstream transcription factor YY1 and RNA binding protein eIF4A3 could facilitate circ-ZNF609 expression by promoting the gene transcription and circRNA circular formation, respectively. These results indicated that circ-ZNF609 might be the novel biomarker of targeted therapy and prognosis evaluation of cholangiocarcinoma.

## MATERIALS AND METHODS

### Patients and clinical specimens

61 pairs of CCA tissues and adjacent normal tissues from patients with CCA treated at The 2nd Affiliated Hospital of Harbin Medical University were obtained after informed consent of the participants and approval of the Ethics Committee of The 2nd Affiliated Hospital of Harbin Medical University. The obtained tissues were rapidly frozen in liquid nitrogen and stored at -80° C for the following studies. All patients did not receive radiotherapy or chemotherapy treatment before operation.

### Cell culture

Normal biliary cell line (HIBEC) and the CCA cells (CCLP-1, TFK-1 and QBC939) were stored in our laboratory. RBE was purchased from Type Culture of Chinese Academy of Sciences (Shanghai, China). The above cholangiocarcinoma cell lines and normal biliary cell were cultured in DMEM (Gibco, Grand Island, USA) with 10% FBS (Invitrogen, Carlsbad, USA) and 1% double antibody (Sigma, St. Louis, USA) in an incubator at 37° C with 5% CO_2_. It was passaged every 72 hours, and all cells were cultured for less than 6 months.

### Cell transfection

All negative controls and the small interfering RNA (siRNA) for circ-ZNF609 (si-circ-1, si-circ-2), LRRC1 (si-LRRC1), YY1 (si-YY1), eIF4A3 (si-eIF4A3); the short hairpin RNA (shRNA) for circ-ZNF609 (sh-circ-ZNF609); the miR-432-5p mimics and miR-432-5p inhibitor; the recombinant pcDNA3.1 plasmids (oe-YY1 and oe-eIF4A3) used for overexpression of YY1 and eIF4A3 were all purchased from GenePharma (Shanghai, China). Tumor cells were transfected using Lipofectamine 3000 reagent (Invitrogen) according to the manufacturer’s protocol. Supplementary Table 1 showed the sequences used in this study.

### Quantitative reverse transcription PCR

Total RNA was extracted from cells and frozen tissue samples using TRIzol reagent (Invitrogen) based on the manufacturer’s protocol and complementary DNA (cDNA) was synthesized using the Transcriptor First Strand cDNA Synthesis Kit (Roche, Penzberg, Germany). FastStart Universal SYBR Green aster (Roche) and C1000 Thermal Cycler (Bio-Rad, Hercules, USA) were used for 10 μl volume qRT-PCR. GAPDH and U6 were used as controls. The 2^-ΔΔCt^ method was used to calculate the transcription level of target genes. RNase R (Epicentre, Madison, USA) was used to degrade linear mRNA. All the primer sequences were listed in Supplementary Table 1.

### Cell viability assay

The cells in logarithmic growth phase were inoculated in 96-well plates at a density of 5×10^4^ cells per well. The cells were cultured for 0-72 h. Every 24 h, 10 ul of CCK-8 reagent (Dojindo, Kumamoto, Japan) was inserted into the petri dish. After incubation for 2 h at 37° C in a humidified incubator, the optical density (OD) at 450nm wavelength was detected by microplate reader (Tecan, Männedorf, Switzerland). The cell proliferation capacity was also detected by 5-ethynyl-2’-deoxyuridine (EdU) assay. The treated cells were inoculated in 96-well plates. After incubation for 24 h, 100 μl EdU solution (Ribobio, Guangzhou, China) was added to each well and stored for 2 h. The treated cells were fixed with paraformaldehyde and then stained with Apollo 567 and Hoechst 33342 in the dark. The EdU-positive cells were detected using a fluorescence microscope (Leica, Wetzlar, Germany). After treatment, cells were diluted and inoculated in 6-well plates at a density of 1×10^3^ cells per well. The treated cells were immobilized with paraformaldehyde and stained with crystal violet (Beyotime, Beijing, China) for 30 minutes. When colonies were visible the transfected cells were preserved in DMEM containing 10% FBS, which were incubated at 37° C with 5% CO_2_ for 2 weeks. The colonies were then fixed with paraformaldehyde and stained with crystal violet (Beyotime, Beijing, China) for 30 minutes. The plates were photographed, and a large number of stained colonies were counted.

### Migration and invasion assays

The CCA cells migration ability was measured by wound healing assay. When the fusion degree of CCA cells plated in 6-well plates reached 80-90%, a straight-line wound was scratched on the surface with ruler and pipette. The percentage of cell healing was measured by taking photographs and calculating the average distance of wound cells at 0 h and 48 h. Transwell assay was performed with transwell chambers (Corning, NY, USA). Transfected cells were placed on the upper chamber at a density of 1×10^5^ cells per well, coated without Matrigel (BD, San Jose, USA) and contained with DMEM (serum-free). And DMEM with 10% FBS was added to the lower chamber. After 24 h inoculation, upper cells were gently removed, and the migrating cells were immobilized, stained with paraformaldehyde and crystal violet respectively, and then the cells were photographed with an inverted microscope (Leica). The invasion assay resembled the migration assay except that the chambers were precoated with Matrigel. The migration and invasion abilities were counted through respectively counting the migrated and invaded cells.

### Western blot assay

The transfected cells were treated with RIPA lysis buffer (Beyotime) and the total proteins were extracted. Protein concentrations were tested with a bicinchoninic acid kit (Beyotime). The protein lysates were split by 12% sodium dodecyl sulfate polyacrylamide gel electrophoresis (SDS-PAGE) and then transferred to polyvinylidene fluoride (PVDF) membrane (Millipore, Billerica, USA). The PVDF membranes were then blocked with 5% skimmed milk powder for one hour, and then incubated with the primary antibody (overnight) and the secondary antibody (1.5 h) in turn. Proteins on the membrane were visualized by BeyoECL kit (Beyotime). GAPDH was applied as an endogenous control and all antibodies were purchased from Abcam (Cambridge, USA).

### Subcellular fractionation assay

Cytoplasmic and nuclear components were separated by Paris kit (Life Technologies, Carlsbad, USA). After lysis of gelatin cells with cell separation buffer, the upper layer of the gelatin cells was centrifuged to obtain the cytoplasmic contents, and the lower layer of the precipitated part was treated with cell division buffer to extract the nuclear contents. The RNA extracted from cytoplasm and nucleus was detected by qRT-PCR. Cytoplasmic control and nuclear control were expressed by GAPDH and U6, respectively.

### Dual luciferase reporter assay

The sequence of circ-ZNF609 and LRRC1 3’-untranslated region (UTR) containing the predicted miR-432-5p binding site was inserted into a firefly luciferase gene reporter vector pmirGLO (Promega, Madison, USA), respectively. The plasmids were synthesized by Invitrogen. The pmirGLO-circ-ZNF609-WT, pmirGLO-circ-ZNF609-MUT, pmirGLO-LRRC1-WT or pmirGLO-LRRC1-MUT were used with miR-432-5p mimics (inhibitor) or NC mimics (inhibitor) (Ribobio) were co-transfected into tumor cells with Lipofectamine 3000 reagent. After 48 h transfection, according to the manufacturer’s guidelines, luciferase was detected though using the dual luciferase reporter detection system kit (Promega).

### RNA immunoprecipitation (RIP) assay

RIP assays were performed using the EZ-Magna RIP RNA-Binding Protein Immunoprecipitation Kit (Millipore) in accordance with the manufacturer’s instructions. The RIP lysis buffer containing protease inhibitor cocktail and RNase inhibitor was made to lyse and then collect cells in different groups. Cells were incubated with RIP buffer containing magnetic beads coated with Ago2 antibodies (Millipore). After incubation at 4° C for 2 h, the coprecipitated RNA was eluted and the extracted RNAs was analyzed by qRT-PCR. IgG group was the negative control group.

### Chromatin immunoprecipitation (ChIP) assay

The interaction between transcription factor YY1 and circ-ZNF609 promoter was verified by EZ-ChIP kit (Millipore) and producer’s recommendations. The cross-linking reaction was performed with 1% formaldehyde, sonication converted the DNA-protein complex into small chromatin fragments. Then IgG and anti-YY1 antibodies (Abcam) were added to form immunoprecipitation. After the cross-link was washed and reversed, qRT-PCR was used to detect the relative enrichment of circ-ZNF609 fragments containing speculated YY1 binding sites in the DNA promoter region.

### Tumor xenograft assay

BALB/c nude mice were purchased from Vital River (Beijing, China) and housed at constant humidity (45-50%) and temperature (25-27° C). 6-week-old nude mice were *in situ* injected with CCA cells in logarithmic phase. The cell suspension 0.2 ml was injected subcutaneously into the groin to construct the model of xenograft of cholangiocarcinoma. 4 weeks later, the mice were sacrificed and the weight of the tumor was weighed by balance. The study was authorized by the Ethics Committee of The 2nd Affiliated Hospital of Harbin Medical University.

### Statistical analysis

GraphPad Prism 8.4 software (GraphPad Software, La Jolla, CA) and SPSS 21.0 software (IBM SPSS, Armonk, NY) were utilized in this study. Measurement data were expressed as mean ± standard deviation (SD) and based on at least three independent experiments. Comparison between cholangiocarcinoma tissues and adjacent normal tissues were performed by paired *t-test*. Data were compared between two groups using an unpaired *t-test*, and between multiple groups using one-way analysis of variance (ANOVA). Pearson correlation was used to analyze the relationship between the two indicators. The survival rate of the patients were calculated using the Kaplan-Meier and Cox regression analysis. The diagnostic value of circ-ZNF609 expression in cholangiocarcinoma was assessed by receiver operating characteristic (ROC). *P* value< 0.05 was indicative of statistically significant difference.

## Supplementary Material

Supplementary Table 1
