# Supplementary material for: YY1 and eIF4A3 are mediators of the cell proliferation, migration and invasion in cholangiocarcinoma promoted by circ-ZNF609 by targeting miR-432-5p to regulate LRRC1
Source: Aging (Albany NY). 2021 Dec 13;13(23):25195–212. doi: 10.18632/aging.203735 (PMC8714144; doi:10.18632/aging.203735)
Supplement: Supplementary Table 1 [file aging-13-203735-s001.pdf]

SUPPLEMENTARY TABLE

Supplementary Table 1. Primer sequences for qRT-PCR and siRNAs sequences.

Nucleic acids	Sequences
GAPDH	F: 5'-GAAGGTGAAGGTCGGAGT-3'<br>R: 5'-GAAGATGGTGATGGGATTTC-3'
U6	F: 5'-GCTTCGGCAGCACATATACTAAA-3'<br>R: 5'-CGCTTCACGAATTTGCGTGTC-3'
ZNF609	F: 5'-TGGTCCACCGTTGTCACTAA-3'<br>R: 5'-ACAGCGAGCTCTGTGGAAAT-3'
circ-ZNF609	F: 5'-GCGCTCAATCCTTTGGGAAC-3'<br>R: 5'-GGACAACATCATTGCTTTTCAGAC-3'
E1	F: 5'-GAAGGATGCCCCAGGATAAG-3'<br>R: 5'-TCCTTCCTGTGTCCTTGACC-3'
E2	F: 5'-TGGGAGGGCTCACTTAAACA-3'<br>R: 5'-TCTCTGATTGCCCAGCATAC-3'
E3	F: 5'-TGAAACCATCAGGAGCTGTG-3'<br>R: 5'-CGTAGGGGATCTGAACATGG-3'
YY1	F: 5'-CTTCCCTTGCCTCTCACAAG-3'<br>R: 5'-GCCGCCTACAGTCTCATAGC-3'
eIF4A3	F: 5'-CGCGGACTCTGACATATGGCGACCACGGCCACGATG-3'<br>R: 5'-TCCCGCAGGCCCATGGTGTC-3'
miR-942	RT: 5'-CTCAACTGGTGTCGTGGAGTCGGCAATTCAGTTGAGCACATGGC-3'<br>F: 5'-ACACTCCAGCTGGGTCTTCTCTGTTTTG-3'<br>R: 5'-TGGTGTCGTGGAGTCG-3'
miR-149	RT: 5'-CTCAACTGGTGTCGTGGAGTCGGCAATTCAGTTGAGGGGAGTGA-3'<br>F: 5'-ACACTCCAGCTGGGTCTGGCTCCGTGTCT-3'<br>R: 5'-TGGTGTCGTGGAGTCG-3'
miR-432-5p	RT: 5'-CTCAACTGGTGTCGTGGAGTCGGCAATTCAGTTGAGCCACCCAA-3'<br>F: 5'-ACACTCCAGCTGGGTCTTGGAGTAGGTCA -3'<br>R: 5'-TGGTGTCGTGGAGTCG-3'
miR-487a	RT: 5'-CTCAACTGGTGTCGTGGAGTCGGCAATTCAGTTGAGCGAACACA-3'<br>F: 5'-ACACTCCAGCTGGGGTGGTTATCCCTGC-3'<br>R: 5'-TGGTGTCGTGGAGTCG-3'
LRRC1	F: 5'-TCCTTACCAAAAGAGATCGG-3'<br>R: 5'-GGTAGATGCAGCAACCTGT-3'
si-NC	5'-TTCTCCGAACGTGTCA-3'
si-circ-1	5'-GTCAAGTCTGAAAAGCAATGA-3'
si-circ-2	5'-AAGTCTGAAAAGCAATGATGT-3'
si-NC	5'-UUCUCCGAACGUGUCACGUTT-3'
si-YY1	5'-CGACGACUACAUUGAACAATT-3'
si-eIF4A3	5'-CGCGAAAGCGGCUGCUCAATT-3'
si-LRRC1	5'-GGAGUGCGAGUUAAAUAAATT-3'
